# Comprehensive evaluation of software system reliability based on component-based generalized G-O models

**DOI:** 10.7717/peerj-cs.1247

**Published:** 2023-02-09

**Authors:** Yuzhuo Wang, Haitao Liu, Haojie Yuan, Zhihua Zhang

**Affiliations:** 1 College of Weaponry Engineering, Naval University of Engineering, Wuhan, Hubei, China; 2 Department of Mathematics, Faculty of Foundation, Naval University of Engineering, Wuhan, Hubei, China; 3 College of Naval Architecture and Ocean Engineering, Naval University of Engineering, Wuhan, Hubei, China

**Keywords:** Component-based software, Fault detection rate, Non-homogeneous Poisson process, The number of remaining faults

## Abstract

The component-based software system has a core that is based on architecture design. Predicting the reliability growth trends of a software system in the early stages of the development process is conducive to reducing waste and loss caused by blind development. Restricted by the lack of information and data in the design and integration phase, it is difficult to implement reliability prediction research at this stage. In this article, we focus on a software system in which the reliability of each component follows the G-O model. First, two system-level parameters, which are the total number of system faults and the detection rate of the system faults, are defined. Then, by studying the relationship between the total number of faults and the detection rate of faults between the components and the system, the defined system parameters are calculated from the known component parameters. On this basis, and by incorporating the system parameters, we construct a reliability growth model for the software system, called the component-based generalized G-O model (CB-GGOM). Besides, two approximate models of CB-GGOM are proposed to facilitate reliability evaluation of the software system in the early and stable stages of the integration test. An engineering explanation of the proposed models is also provided, and their effectiveness is verified through simulation and with an authentic example. Since the proposed models are formulated without any integration test data, they are beneficial for developers to optimize test strategies of the software system and implement defect prevention in advance.

## Introduction

With the development of software integration techniques, component-based software engineering (CBSE), which is a technique for software reuse, has become widely used in the development of software systems. The goal of CBSE is to construct a new software system by integrating several existing components that are connected loosely and interact with each other in an interoperable way (Jha & Mishra, 2019). The concept of CBSE is to reduce the difficulty of software development, save development time and costs, and improve the autonomy and maintainability of components through component reuse. This concept effectively meets the developmental needs of large-scale software systems in an open environment (Phani Krishna et al., 2021).

At present, the scale of component-based software systems is becoming larger, and the applications of this type of system in aviation, military, medicine, and other key fields are constantly growing (Lindoerfer & Mansmann, 2017; Park & Choi, 2018; da Silva et al., 2022; Slabinoha et al., 2021). However, a small bug may lead to the malfunction of components and even the failure of the entire system, leading to disastrous consequences (Aydin, 2020; Fitzgerald, 2012). Therefore, as an essential metric of software quality, the reliability of component-based software systems has attracted much attention from academia and the software industry.

To evaluate and predict the reliability level of software, traditional analysis methods usually regard software as a black box and establish software reliability growth models (SRGMs) by collecting the fault data of software in the testing or operation stage. These black box methods mainly applied to early small-scale software and were the prominent approach for software reliability evaluation until the early 21st century (Ce et al., 2017). However, the reliability of component-based software system is related to component reliability and is also affected by system architecture, component interface reliability, and the way the system is operated by users (Bistouni & Jahanshahi, 2020). Moreover, components are usually tested before delivery, which makes the fault data of the software system a small sample. Traditional black box methods cannot expose the internal design defects of software system, and require a large number of fault data. Thus, the method of comprehensively evaluating the reliability of software systems by applying component reliability characteristics and system architecture has become an important research area.

The lifecycle of component-based software systems generally includes the stages of analysis/design, implementation/acquisition, integration, test, maintenance/distribution, and administration/support. Also, software architecture design forms the core of the software system. For such a system, the cost of making modifications in the later development stages is massive, while some designs cannot be modified at all. In some specific software system development projects, such as the development of extremely large systems, the core architecture of the system needs to be designed at the outset. Based on passing the test of the core architecture, peripheral components are gradually added to it. To reduce waste and loss caused by blind development, evaluating the reliability of software system in the upstream stage of development has become the focus of many scholars in the field of component-based software system reliability evaluation (Nagarkoti & Charaya, 2019). However, most results from existing research in this field are implemented in the integration test stage or the operation stage, which has rich test data. Restricted by the lack of information and data in the design and integration phases, there are few reliability prediction models for software systems that are applied at these stages.

This article aims to infer the reliability growth trend of a software system during integration testing by using available system architecture and the historical reliability growth trends of components. For this purpose, we focus on a software system in which the reliability of components follows the Goel & Okumoto model (G-O model) (Goel & Okumoto, 1979). First, two system-level parameters are defined, these being the total number of system faults and the detection rate of system faults. Next, by establishing a relationship between the total number of faults and the detection rate of faults in both the components and the system, the defined system parameters can be calculated from the known component parameters. On this basis, by incorporating system parameters, a reliability growth model for software system is constructed and is known as the component-based generalized G-O model (CB-GGOM). For convenience, two approximate models of CB-GGOM are provided, which are respectively applied to reliability evaluations of the software system in the early and stable stages of integration testing. The contributions of this article are as follows: 1. An engineering explanation of the proposed models is given, revealing that the fault detection level of the software system is related to the test time spent on each component and also associated with the number of remaining faults in the component that is currently executed; 2. the proposed models can be calculated without any integration test data, so it is beneficial for developers to optimize test strategies of the software system and implement defect prevention in advance.

The remainder of this article is organized as follows: Section 2 introduces research work in related fields and the research motivation of this article. In Section 3, we analyze the available information before system integration and propose some basic assumptions. In Section 4, the estimation models of two system parameters and the reliability growth models of the system are proposed. Also, the engineering interpretation is provided. Section 5 verifies the effectiveness of the proposed models through simulation and authentic example. Section 6 discusses the applications of the proposed models. Section 7 discusses the threats to the validity of the proposed models and future work.

## Related works and research motivation

At present, there are several reliability evaluation models for component-based software systems that can be divided into static and dynamic models.

Static reliability evaluation models generally focus on the modeling of system architecture under the assumption that component reliabilities are known, thereby obtaining the point estimation of software system reliability. These models can be further categorized into path-based methods and state-based methods. Path-based methods employ all executed paths to model the system architecture and apply the reliabilities of all components in the path to represent path reliability. Then, they utilize the path reliabilities and path execution probabilities to calculate overall system reliability (Hsu & Huang, 2011; Nautiyal, Gupta & Dimri, 2019). State-based methods usually map the system architecture into a state space model, such as the discrete-time Markov chain (DTMC) (Cheung, 1980), continuous-time Markov chain (Jean-Claude, 1984), or semi-Markov process (Littlewood, 1979). They utilize the transition of state to represent the interactive transition between components, then use the transition probabilities and reliabilities of components to determine the reliability of the software system. However, as integration testing and system testing proceed, internal component faults and component interface faults are continually detected and removed. Static evaluation methods cannot solve system reliability growth issues generated by the continuous growth of errors.

To achieve time-dependent system reliability evaluation, some dynamic evaluation methods, such as the dynamic probability transition graph (Mao & Yongjin, 2004) and stochastic Petri net (Wang, Lei & Han, 2015), have been proposed by extending the static evaluation models. Software systems connected by loose coupling may still have interaction dependency during actual operation. Therefore, some scholars have proposed dynamic evaluation models of system reliability that consider component dependency on the premise that all test data is monitored (Mehra & Kapil, 2020; Tiwari, Kumar & Matta, 2020). Accordingly, the evaluation results have reference value for improving component reusability. Since the above models need to track the testing process, their calculations either require the fault data of components in the operation process, the interaction times of components, or the times of paths selected. These data are mainly obtained by statistics or simulation, so they are applied in the integration test stage or the operation stage.

To shorten the reliability evaluation time, Shanmugaiah & Ayyaswamy (2019) proposed a system reliability prediction framework based on the graph theory. This framework did not exhaust all test paths, so it saved test time while ensuring evaluation accuracy. Talafuse & Pohl (2018) used the grey prediction model to evaluate the system reliability under the constraint of small samples. Yang (2012) improved the accuracy of the grey prediction model by applying the genetic algorithm. However, the grey prediction model needs to generate and expand valuable information based on known information, so the evaluation methods that incorporate small sample technology and intelligent algorithms can still be used after the integration test begins.

Component-based software reliability growth models (CB-SRGMs) are models that focus on the growth process of software system reliability. They generally use SRGMs to model the growth processes of component reliabilities, thereby establishing a relationship between components and the software system. Subsequently, they evaluate and predict system reliability. The function of the cumulative number of system faults is the kernel of CB-SRGMs. Additive models are representative CB-SRGMs. By assuming that the fault process of each component followed the non-homogeneous Poisson process (NHPP) and the system had a serial architecture, Xie & Wohlin (1995) proposed the earliest additive model, where the cumulative fault function of the system equaled the sum of the corresponding functions of its components. Obviously, the Xie model did not explicitly take the system architecture into account, nor did it consider the difference between the test times spent on each component. This means that all components were constantly activated. Given the above-mentioned issues, Chunyan et al. (2009) modeled the system architecture using the DTMC and introduced a proportional time factor to represent the time spent on the components. This improved the additive relationship of cumulative fault function between the components and the system. Afterward, Jain, Jain & Gupta (2018) took software test costs and optimal software release time into account. However, in the case of a large number of components, too many summation terms make the expression of the additive models too complex, and interpretability is also lacking. Under the assumption that the reliability of each component followed the G-O model, Chunyan et al. (2009) proposed a reliability growth model for software system and named it the component-based non-homogeneous Poisson process (CB-NHPP) model. The CB-NHPP model applied the expression of the G-O model, had interpretable parameters, and could be performed without integrating test data. But as far as the follow-up experimental results show, its evaluation effect is not satisfactory.

In general, although there are many reliability evaluation methods for component-based software systems, the guiding ideology of most studies is to simplify the models as much as possible to reduce time and cost while ensuring evaluation accuracy. However, most existing methods are employed in the integration test stage or operation stage, when test data are abundant. Restricted by the lack of information and data in the design and integration stage, few system reliability prediction models are applied at these stages, and the evaluation and prediction performances are not satisfactory.

A new system assembled from components is substantially different from single software developed from scratch since each component has historical test information. This information can be used to establish SRGMs that reflect historical fault trends of components. Integration tests have not been carried out, so any data generated during system operation cannot be obtained. However, the system architecture is designed in advance and the integration test is usually performed by simulating the operating behavior of users. This means that random interaction in integration testing should follow a certain statistical regularity. Therefore, we can utilize the known system architecture and historical fault trends of the components to infer the fault trends of the software system during the integration testing process. The NHPP is the most popular theory for describing the fault trend of a single software, while the G-O model has important theoretical value. Because of the above considerations, we intend to conduct research under the assumption that component reliability follows the G-O model.

## Basic assumptions

Components can be diversely defined depending on the discipline (van Driel, Fan & Zhang, 2018). In the field of software engineering, the representative definition of the term “component” was given by Szyperski, Bosch & Weck (1999): “a component is a combined unit with certain interface specifications and obvious environment interaction, without an externally visible state, which can be independently configured and easily integrated by a third party.” They are usually encapsulated according to their function, developed by independent teams, and also sometimes written in different languages. Therefore, components are a kind of black box with high cohesion and low fault correlation between them. They are connected in a loosely coupled manner and there may be interaction dependencies in the actual running environment. However, excessive interaction dependencies are controlled and minimized by engineers to achieve component reusability (Ruiz Ceniceros et al., 2021). Therefore, we can assume that the fault processes between components are independent, and the interaction processes between components are also independent; to ensure component quality, the components should undergo unit testing before delivery. The unit testing environment is independent of other parts of the software system, so individual component faults are centrally removed while growing fault data is accumulated at this stage. These data are generally utilized to establish SRGMs and evaluate the reliability level of components, so the SRGMs of the components in the unit test stage are considered known; before integration, the architecture of the new system is available and it predefines the connection mode between components. Connection of the components must be realized through component interfaces. The component interfaces are usually explicit codes that describe the import and export relationships between components, and act as independent components (Hamlet, Mason & Woit, 2001). Therefore, a component interface with a simple architecture can be regarded as completely reliable. On the other hand, a component interface with complex architecture can be split off, analyzed, improved, and then assembled with other components (Garg & Lai, 2018).Thus, we can regard the component interfaces as components themselves and use the SRGMs to describe their reliabilities; after integration, developers must test the new system to eliminate interface faults and verify the functions of components and the new system. For large-scale software systems, developers usually adopt integration testing based on an operation profile. The operation profile is a model that describes how software may be used in an expected operating environment, including the users’ expected operation of each functional module and the probability of each operation (Musa, 1993). In integration testing based on the operation profile, the calling of components is performed according to the operation profile, which is a white box testing process. Each activated component is subjected to black box testing, and the testing environment is similar to the continuation of its previous unit testing. So, the overall integration testing process is a gray box testing process that simulates the operation of users. Construction of the operation profile model is generally a necessary expense in the development cost of large-scale software systems. Existing operation profile construction methods include the expert experience method, early version method, and simulation method (Amrita & Kumar, 2017; Fiondella & Gokhale, 2012; Pietrantuono, Popov & Russo, 2020). In this article, we will not discuss the specific technology, but only assume that the operation profile model is known and reasonably constructed. Therefore, the following assumptions are proposed:

1) The initial time of the software system integration is 0.
2) The fault process of the component follows the NHPP. The component fault trend in the unit test stage can be modeled using SRGM, which is known and is assumed as the G-O model (Goel & Okumoto, 1979), *i.e*.:

(1) $${m_i}(t) = {a_i}[1 - \exp ( - {b_i}t)],0 \le t \le {T_i}$$

where *T*_*i*_ is the last unit testing fault time of component *i*, *m*_*i*_(*t*) represents the expected number of faults detected from component *i* when the unit testing time reaches *t*, and once a fault is detected during unit testing, it immediately causes component failure. The constant *a*_*i*_ denotes the total number of faults in component *i*, and the constant *b*_*i*_ represents the fault detection rate of component *i* in the unit testing stage. Here, *a*_*i*_ and *b*_*i*_ are estimated through unit testing fault data.

3) The component interfaces are considered to be completely reliable. A larger-scale component interface can be abstracted as a separate component, and its reliability follows the G-O model with known parameters, while the remaining smaller-scale component interface is assumed to be simple enough.
4) The fault processes between components are independent, and the interaction processes between components are also independent. The operation profile model is known.

**Figure 1:**
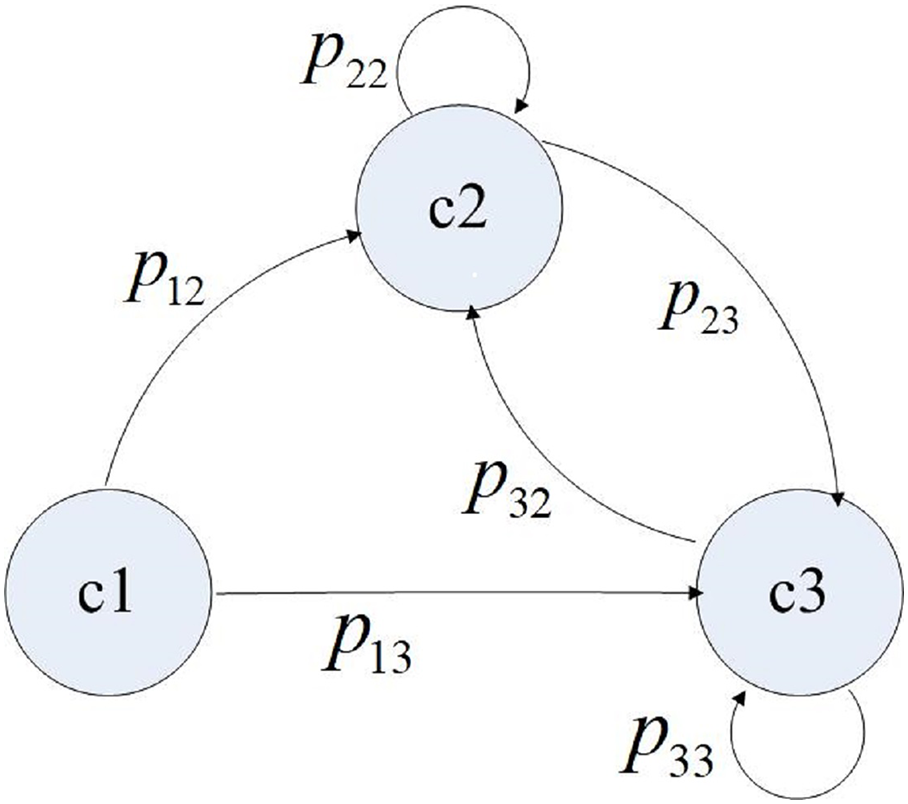
Control transition of a simple software application.

Next, we define *I* = {*i*|*i* = 1,2,…,*n*} as a discrete set of states, where element *i* represents the *i*^*th*^ component, including the component interface. Also, (0, *s*) is the continuous operation time interval between any two system failures, while the random variable *v*_*i*_ is the residence time of the system on the active component *i*. *S* = {*s*_1_,*s*_2_,…,*s*_*m*_,…} is the set of transition moments during system operation, which satisfies 0 < *s*_1_ < *s*_2_ < …< *s*_*m*_ <…< *s*. Also, *X*(*s*_*m*_) = *i* is the component activated after the *m*^*th*^ operation transition. *P*{*X*(*s*_*m+*1_) = *j|X*(*s*_*m*_) = *i*} = *p*_*ij*_ is a one-step transition probability from component *i* to component *j* at the *m*+1^*th*^ operation transition, and *P* is the transition probability matrix. Based on the above assumptions, the state transition process at each operation transition time has no aftereffect. Thus, it can be described by a time-discrete Markov chain, which is time-homogeneous. The value of *p*_*ij*_ is determined by the architecture design of the software system and the operating behavior of users. If component *i* and component *j* are designed not to interact directly, *p*_*ij*_ is assigned as 0. Otherwise, the value of *p*_*ij*_ is given by the operation profile model. Thus, the control transition of a simple software application is shown in Fig. 1 (Control transition of a simple software application).

## Software system reliability growth model

When the interaction processes of components are assumed to be completely independent and the fault process of each component follows the NHPP, then the entire fault process of the system is an independent incremental process that can also be described using the NHPP (Everett, 1999; Xie & Wohlin, 1995). This feature leads us to a modeling idea: no matter how complicated the interaction within the software system is, its entire fault process still follows NHPP, thereby enabling us to define the total number and detection rate of system faults, then construct the SRGM of the software system by incorporating the system parameters. However, the traditional parameter estimation method is not adopted by us because it is mainly aimed at software without historical information. Only when the software is tested and sufficient fault data are collected can the parameters be estimated, which makes the corresponding SRGM only applicable in the later stages of testing. In this section, we propose two estimation models for the defined system parameters that can be calculated from known component parameters. In this way, the SRGM of a software system with such parameters can be used in the design and integration stages.

### Estimation model for the total number of system faults

The total number of software faults refers to the sum of the cumulative number of faults that have been removed when software testing reaches time *t* and the number of remaining latent faults in the software has not yet been detected. When the reliability of each component follows the G-O model, the total number of faults in the component is assumed to be constant and no new faults are introduced during the debugging process. Therefore, the total number of faults of the entire software system should also be constant, which can be written as the notation *A*_*s*_. The relationship between *A*_*s*_ and *a*_1_, *a*_2_, …, *a*_*n*_ is written as a function of *f*.

Because the value of *A*_*s*_ does not change as integration testing proceeds, it is considered equal to the sum of the total number of faults in each component at the start of integration. Each component is tested before integration with many internal faults already having been removed. Therefore, if ${\bar {\rm m}_{\rm i}}{\rm (t)}$ represents the number of remaining faults in component *i* when unit testing reaches time *t*, ${\bar {\rm m}_{\rm i}}{\rm (}{{\rm T}_{\rm i}}{\rm )}$ equals the total number of faults in component *i* at the initial time of integration. This satisfies:

(2) $${\bar m_i}({T_i}) = {a_i} - {m_i}({T_i})$$

where *T*_*i*_ is the final unit testing fault time of component *i*. Thus, *A*_*s*_ can be expressed as:

(3) $${A_s} = \sum\limits_{i = 1}^n {{{\bar m}_i}({T_i})}$$

By substituting the *m*_*i*_(*t*) from Eq. (1) into Eqs. (2) and (3), we obtain a model for function *f*, which is the estimator of *A*_*s*_:

(4) $${\hat A_s} = f({a_1},{a_2},...,{a_n}) = \sum\limits_{i = 1}^n {[\exp ( - {b_i}{T_i}} )] \cdot {a_i}$$

Equation (4) indicates that the total number of faults in the software system is a linear addition of the number of faults in each component. Here, the weight exp(−*b*_*i*_*T*_*i*_) measures the sufficiency of component *i* being tested in the unit test phase.

### Estimation model for the fault detection rate of the software system

The fault detection rate (FDR) describes the efficiency of software fault detection in the current testing environment. Because the testing process of the software system is considered a control transition process, the FDR of the system at time *t* is closely related to the FDR of the component currently being executed and also changes with the transition of the executed component. Thus, we consider the FDR of the system as a function of time and express it as *B*_*s*_(*t*). Moreover, the relationship between *B*_*s*_(*t*) and *b*_1_, *b*_2_, …, *b*_*n*_ is a function *g*.

When the fault process of the software follows the NHPP, it is regarded as having a fault intensity function during the fault process. Mathematically, the software FDR can be defined as the ratio of the software fault intensity function to the number of remaining software faults at time *t* (Pham, Nordmann & Zhang, 1999). This represents the probability that each remaining software fault is detected. Based on previous assumptions, the entire fault process of the software system follows the NHPP. Thus, the FDR of the software system can be defined as:

(5) $${B_s}(t) = \displaystyle{{{\Lambda _s}(t)} \over {{A_s} - {m_s}(t)}}$$

where Λ(*t*) and *m*_*s*_(*t*) respectively denote the fault intensity function and the cumulative fault function of the software system at time *t*. Besides, according to NHPP theory, Λ(*t*) = *m′*_*s*_(*t*). Since *A*_*s*_ has already been expressed by the component parameters, if a relationship between the cumulative fault function of the system and its components can be subsequently established, *B*_*s*_(*t*) can also be expressed by *b*_1_, *b*_2_,…, *b*_*n*_ and the model of function *g* is automatically obtained.

Obviously, *m*_*s*_(*t*) should equal the sum of the cumulative fault functions of the components when integration testing reaches time *t*. However, the components are no longer continuously activated but are instead intermittently exchanged during integration testing. Meanwhile, the function *m*_*i*_(*t*), which is created in a unit testing environment, cannot be directly used in the integration testing phase. To measure the execution state of the component in (0, *t*), we first regard *n* components as *n* states, then use the transition probability matrix *P* to obtain a steady-state distribution of the *n* states, which we express as ${\vec \eta = [}{{\eta }_{\rm i}}{\rm ]}$. This is the solution of ${\vec \eta = \vec \eta }\cdot {\it P}$ and $\sum {\vec \eta = 1}$. Therefore, ${\vec \eta }$ represents the frequency of each component being called in the steady-state; next, we write the notation *τ*_*i*_ as the expectation of the random residence time *v*_*i*_, that is *Ev*_*i*_ = *τ*_*i*_. Thus, *τ*_*i*_ represents the average task time of component *i* in one execution. Its value is determined by the functional attributes of the component and can be obtained from the statistics of unit testing data. Consequently, the factor:

(6) $${\pi _i} = \displaystyle{{{\eta _i}{\tau _i}} \over {\sum\limits_{i = 1}^n {{\eta _i}{\tau _i}} }}$$

can be used to measure the proportion of time spent on component *i* during the stable state of the system (Chunyan et al., 2009). Owing to sufficient unit testing of each component, system failures during integration testing only occur infrequently. That is, the times between system failures are often much longer than that between control exchanges, and many control exchanges would take place between successive system failures. Therefore, the software system can run to a stable state before failure occurs, the proportion of time spent on component *i* in any integration testing phase can be approximately measured by the constant *π*_*i*_. Thus, *m*_*s*_(*t*) can be modeled as follows:

(7) $${m_s}(t) = \sum\limits_{i = 1}^n {[{m_i}({T_i} + {\pi _i}t) - {m_i}({T_i})} ]$$

By substituting the G-O model into Eq. (7), *m*_*s*_(*t*) can be estimated as:

(8) $${\hat m_s}(t) = \sum\limits_{i = 1}^n {{a_i}{e^{ - {b_i}{T_i}}}} (1 - {e^{ - {b_i}{\pi _i}t}})$$

Finally, by integrating Eqs. (4) and (8) into Eq. (5), the estimator of *B*_*s*_(*t*) is:

(9) $${\hat B_s}(t) = \sum\limits_{i = 1}^n {\left[ {\displaystyle{{{a_i}{e^{ - {b_i}({T_i} + {\pi _i}t)}}} \over {\sum\limits_{i = 1}^n {{a_i}{e^{ - {b_i}({T_i} + {\pi _i}t)}}} }}{\pi _i}{b_i}} \right]}$$

If we write the factor *ω*_*i*_(*t*) as:

(10) $${\omega _i}(t) = \displaystyle{{{a_i}{e^{ - {b_i}({T_i} + {\pi _i}t)}}} \over {\sum\limits_{i = 1}^n {{a_i}{e^{ - {b_i}({T_i} + {\pi _i}t)}}} }}$$

the estimator of ${\hat {\rm B}_{\rm s}}{\rm (t)}$ can be shown as:

(11) $${\hat B_s}(t) = g({b_1},{b_2},...,{b_n}) = \sum\limits_{i = 1}^n {{\pi _i}[{\omega _i}(t)} \cdot {b_i}]$$

### Engineering interpretation of ${\hat {\rm B}_{\rm s}}{\rm (t)}$

By observing Eq. (10), it is apparent that *ω*_*i*_(*t*) is the proportion of the remaining faults in component *i* to the remaining faults in the software system at time *t*. Therefore, if we name *ω*_*i*_(*t*) the proportional factor of the remaining faults in component *i*, then Eq. (11) can be explained like this: *b*_*i*_ is estimated according to the component fault data generated in the unit test stage. It is a known parameter that reflects the historical fault detection level of component *i*; the fault detection level of component *i* in the integration test stage is unknown, for ease of expression, we use the notation *b*_*i*_^*IT*^(*t*) to represent it. Therefore, according to Eq. (11), *ω*_*i*_(*t*)*b*_*i*_ is the prediction for *b*_*i*_^*IT*^(*t*); because integration testing is a process of control transition, each component is not allocated the entire integration testing time but is assigned a proportion of the total test time as *π*_*i*_. Thus, the weighted sum of the probability of detecting faults in each component within (0, *t*) and its actual contribution proportion to the system is an estimation of the system fault detection probability within (0, *t*). We can imagine that without *π*_*i*_, the test of the software system is a parallel test rather than a control transition process. Also, if there is no *ω*_*i*_(*t*) but only *b*_*i*_ to predict *b*_*i*_^*IT*^(*t*), the test of the software system then becomes a process of *n* newly developed components being independently tested. In such a testing environment, the number of initial faults in each component is the largest, each component is on an independent testing platform with its own exclusive testing resources, and the test for each component is continuous, just the proportion of tested time is different. However, every component entering the integration testing stage has been tested, so actually there are few residual faults; moreover, these faults are no longer concentrated in a single component but are unevenly distributed within each component. With the transition of system operation, *n* components share a set of test resources. A reduction in the number of latent faults and changes in the distribution of latent faults are the main differences between the two test stages of the components. The meanings of “remaining faults” and “proportion” of *ω*_*i*_(*t*) reflect the above component changes from unit testing to integration testing. Therefore, using *ω*_*i*_(*t*)*b*_*i*_ to predict the fault detection level of component *i* in the integration test stage is a correction of prediction as *b*_*i*_.

To further investigate the rationality of ${\hat {\rm B}_{\rm s}}{\rm (t)}$, we focus on its values in two extreme states. At the initial moment of integration testing, the value of ${\hat {\rm B}_{\rm s}}{\rm (t)}$ equals:

(12) $${\hat B_s}(0) = \sum\limits_{i = 1}^n {{\omega _i}(0){\pi _i}{b_i}}$$

where *ω*_*i*_(0) can be calculated as:

(13) $${\omega _i}(0) = \displaystyle{{{a_i}{e^{ - {b_i}{T_i}}}} \over {\sum\limits_{i = 1}^n {{a_i}{e^{ - {b_i}{T_i}}}} }}$$

At this time, the number of remaining faults in the software system is the largest, so the fault detection ability of the system is strong. As testing proceeds, the weight *ω*_*i*_(*t*) continuously fluctuates. When the testing enters a stable state, the limit of ${\hat {\rm B}_{\rm s}}{\rm (t)}$ can be calculated as:

(14) $${B_s}(\infty ) = \mathop {\lim }\limits_{t \to \infty } \displaystyle{{\sum\limits_{i = 1}^n {{\pi _i}{b_i} \cdot {a_i}{e^{ - {b_i}{T_i}}}{e^{ - {\pi _i}{b_i}t}}} } \over {\sum\limits_{i = 1}^n {{a_i}{e^{ - {b_i}{T_i}}}{e^{ - {\pi _i}{b_i}t}}} }}$$

We define *B*^(*l*)^_min_ = min(*π*_1_*b*_1_,*π*_2_*b*_2_,*…*,*π*_*n*_*b*_*n*_), where the superscript *l* indicates that the minimum *π*_*i*_*b*_*i*_ is generated by the *l*^*th*^ component. Thus, Eq. (14) can be modified to:

(15) $$\eqalign{
  & {B_s}(\infty ) = \mathop {\lim }\limits_{t \to \infty } {{{e^{{B_{{{\min }^{(l)}}}} \cdot t}}} \over {{e^{{B_{{{\min }^{(l)}}}} \cdot t}}}} \cdot {{\sum\limits_{i = 1}^n {{\pi _i}{b_i} \cdot {a_i}{e^{ - {b_i}{T_i}}}{e^{ - {\pi _i}{b_i}t}}} } \over {\sum\limits_{i = 1}^n {{a_i}{e^{ - {b_i}{T_i}}}{e^{ - {\pi _i}{b_i}t}}} }}  \cr 
  & \quad \quad \quad  = \mathop {\lim }\limits_{t \to \infty } [{\pi _1}({b_1} \cdot 0) + ... + {\pi _l}({b_l} \cdot 1) + ... + {\pi _n}({b_n} \cdot 0)] = {B_{{{\min }^{(l)}}}} \cr} $$

and we can obtain the limit of ${\hat {\rm B}_{\rm s}}{\rm (t)}$:

(16) $${B_s}(\infty ) = \min ({\pi _i}{b_i}),i \in I$$

Now we can explain the FDR of the system in a steady state. When the integration test enters the steady state, the internal faults of almost every component are eliminated and only the component with the slowest fault detection has undetected faults. At this stage, the system still activates the components according to the operation profile, but it is impossible to detect faults in the components that have no faults. After the task is completed, the system quits the active component. Therefore, the FDR of the system is reflected entirely by the calling frequency and the FDR of the last faulty component. Equation (15) shows that under the correction *ω*_*i*_(*t*), the probability of detecting faults in a component without fault tends to zero, and the fault detection rate of the system is equal to min(*π*_*i*_*b*_*i*_).

### Cumulative fault function and reliability function of the software system

The task of establishing a software reliability growth model generally includes modeling the cumulative fault function and the reliability function. When the fault process of the software obeys the NHPP, the latter can be fully represented by the former. In this section, according to the concept of engineering approximation, we model the cumulative fault function and reliability function for the component-based software system by incorporating the system-level parameters proposed in the previous section.

The G-O model uses a negative exponential structure to express the cumulative fault function, so it is also known as an exponential SRGM. Since many SRGMs are derived from the G-O model and essentially belong to the exponential category, the G-O model is considered a model with good flexibility and adaptability. When the reliability of each component follows the G-O model, the debugging process of each component is assumed to be an instantaneous process without the introduction of any new faults. Based on previous assumptions, the debugging process of the system is the same as that of the components. Thus, we can use a negative exponential structure to express the cumulative fault function of the software system. It can be modeled as:

(17) $${\tilde m_s}(t) = {A_s}\{ 1 - \exp [ - {B_s}(t) \cdot t]\}$$

Therefore, if the last startup time of the software system is *t*, then the continuous duration of operation after startup is *s*. According to the definition of software reliability, the probability is that the software will run continuously for a given period without fault. The reliability of the software system within (*t*, *t* + *s*) can be expressed as:

(18) $$\eqalign{ R(s|t) & = \displaystyle{{{{[{{\tilde m}_s}(s + t) - {{\tilde m}_s}(t)]}^0}} \over {0!}}{e^{ - [{{\tilde m}_s}(s + t) - {{\tilde m}_s}(t)]}} \cr & = \exp \{ - [{{\tilde m}_s}(s + t) - {{\tilde m}_s}(t)]\} }$$

Since ${{\tilde {\rm m}}_{\rm s}}{\rm (t)}$ has the same exponential structure as the G-O model, the parameter *B*_*s*_(*t*) is a function rather than a constant. Therefore, Eq. (17) can be regarded as a flexible G-O model with time-varying FDR. With this in mind, we give Eqs. (17) and (18) the joint name of the component-based generalized G-O model (CB-GGOM), where the values of parameters *A*_*s*_ and *B*_*s*_(*t*) are estimated by the component parameters.

Since ${\hat {\rm B}_{\rm s}}{\rm (t)}$ varies with time, the expression of ${{\tilde {\rm m}}_{\rm s}}{\rm (t)}$ given by Eq. (17) remains complicated. However, as the reliability growth process of integration testing is relatively slow, fluctuations of ${\hat {\rm B}_{\rm s}}{\rm (t)}$ over a short time interval are not obvious. If we take any time *ξ* in the interval (*t*, *t* + *s*) and use the corresponding constant *B*_*s*_(*ξ*) to replace the function ${\hat {\rm B}_{\rm s}}{\rm (t)}$, then an approximate model of CB-GGOM within (*t*, *t* + *s*) can be obtained:

(19) $${m_s}(t) \approx {A_s}\{ 1 - \exp [ - {B_s}(\xi ) \cdot t]\}$$

Eq. (19) presents a simple G-O model, meaning that it may be more concise and practical for system reliability evaluation when the evaluation accuracy requirements are less strict. Furthermore, we assume that *ξ* is either 0 or ∞, and consider two special cases of Eq. (19) for the early and stable phases of integration testing. For the sake of brevity, we abbreviate these approximate models as CB-GGOM-E and CB-GGOM-S, respectively. All of our proposed models are listed in Table 1, while the Xie model (Xie & Wohlin, 1995) and the CB-NHPP model (Chunyan et al., 2009) are also listed for comparison.

**Table 1:** Comparison between models.

	Total number of system faults	Fault detection rate of system	Cumulative faults function of system
Xie model	-------------	-------------	$\sum\limits_{i = 1}^n {{a_i}[1 - \exp ( - {b_i}t)]}$
CB-NHPP model	$\sum\limits_{i = 1}^n {{a_i}{e^{ - {b_i}{T_i}}}}$	$\sum\limits_{i = 1}^n {{\pi _i}{b_i}}$	$(\sum\limits_{i = 1}^n {{a_i}{e^{ - {b_i}{T_i}}})} \cdot [1 - \exp ( - \sum\limits_{i = 1}^n {{\pi _i}{b_i}} t)]$
CB-GGOM	$\sum\limits_{i = 1}^n {{a_i}{e^{ - {b_i}{T_i}}}}$	$\sum\limits_{i = 1}^n {{\pi _i}[{\omega _i}(t)} {b_i}]$	$(\sum\limits_{i = 1}^n {{a_i}{e^{ - {b_i}{T_i}}}} ) \cdot \{ 1 - \exp [ - \sum\limits_{i = 1}^n {{\pi _i}{\omega _i}(t)} {b_i}t]\}$
CB-GGOM-E	$\sum\limits_{i = 1}^n {{a_i}{e^{ - {b_i}{T_i}}}}$	$\sum\limits_{i = 1}^n {{\pi _i}[{\omega _i}(0)} {b_i}]$	$(\sum\limits_{i = 1}^n {{a_i}{e^{ - {b_i}{T_i}}}} ) \cdot \{ 1 - \exp [ - \sum\limits_{i = 1}^n {{\pi _i}{\omega _i}(0)} {b_i}t]\}$
CB-GGOM-S	$\sum\limits_{i = 1}^n {{a_i}{e^{ - {b_i}{T_i}}}}$	$\min ({\pi _i}{b_i})$	$(\sum\limits_{i = 1}^n {{a_i}{e^{ - {b_i}{T_i}}}} ) \cdot \{ 1 - \exp [ - \min ({\pi _i}{b_i}) \cdot t]\}$

## Experiments

In this section, we verify the effectiveness of the proposed models through simulation and by applying the models to an example. The significance of proposed models for the early evaluation of software system reliability is also demonstrated.

### Simulation verification

Rate-based discrete-event simulation (RBDES) is an important reliability simulation technique that is applied to individual software. It simulates the random fault behavior of software based on rate-driven event process theory (Olaniyan, Oladosu & Orioke, 2017; Kapur, Aggarwal & Kaur, 2011). By combining RBDES with various system architecture models, many component-level and system-level fault process simulation approaches have been developed. The simulated system fault datasets generated by these new approaches provide a novel method for verifying CB-SRGMs. By combining the RBDES technique with the DTMC model, reference (Wang, 2012) proposed a fault process simulation approach for a software system that had component reliability obeying the G-O models. In this part, we verify the effectiveness and illustrate the significance of the proposed models based on reference (Wang, 2012).

**Figure 2:**
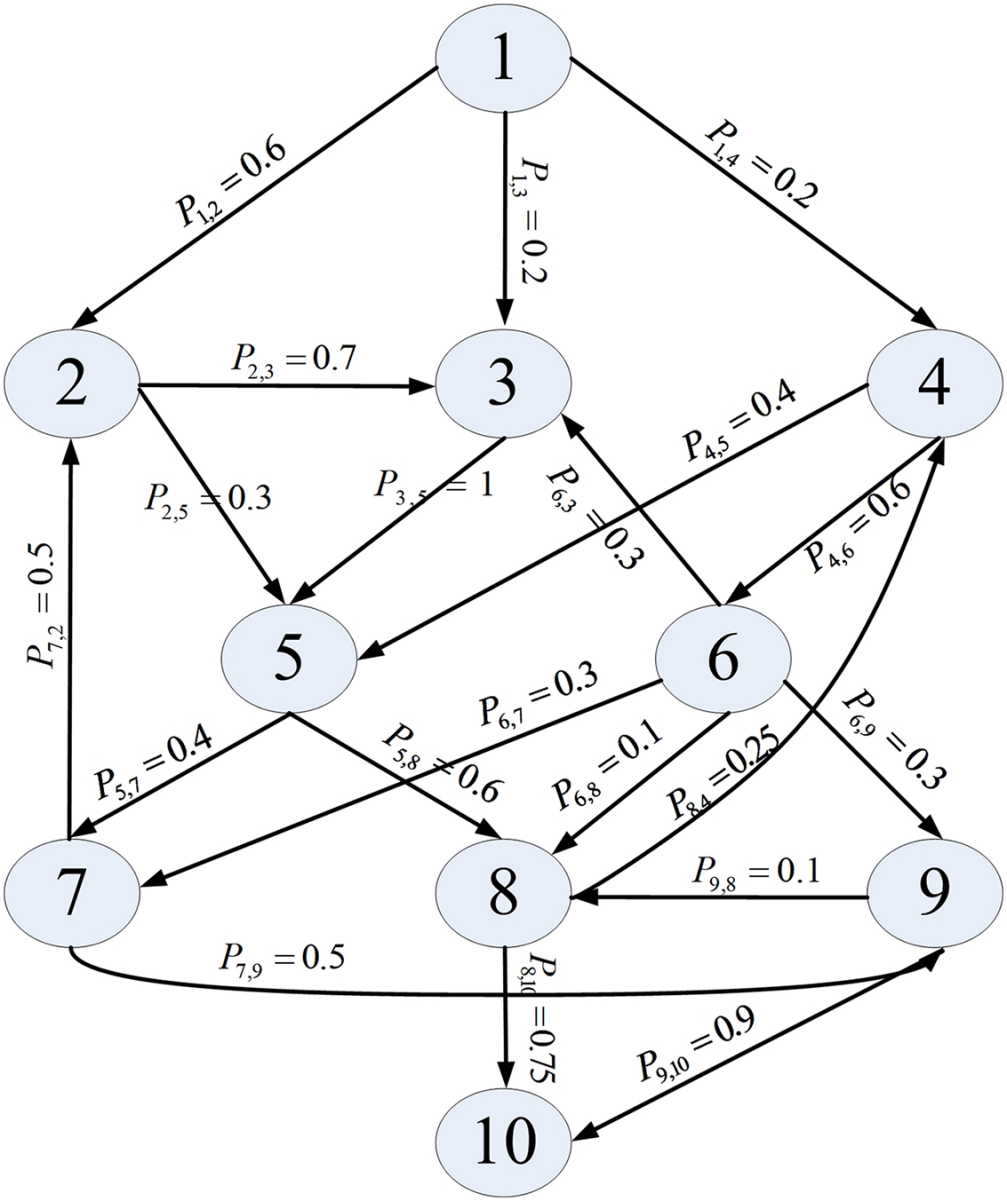
Architecture of an application.

We utilized the software application reported in Cheung (1980) as the simulated object, as it is widely cited in the field of component-based software reliability. This application consists of ten components and its architecture is presented in Fig. 2. Assuming that the component reliability follows the G-O model, we set the parameter values and component unit testing information, as Table 2 shows.

**Table 2:** Component information before assembly.

Component	Average task time in one execution *τ*_*i*_ (unit of time)	Last failure time of unit testing *T*_i_ (unit of time)	Parameter estimate of the G-O model	
			*a*	*b*
*c*_1_	33	80,000	30.0043	0.00002646
*c*_2_	7	76,000	27.8741	0.00001709
*c*_3_	42	85,000	29.9581	0.0000262
*c*_4_	10	83,000	30.2097	0.00002775
*c*_5_	25	90,000	27.9501	0.00001883
*c*_6_	9	83,000	41.8076	0.00003782
*c*_7_	31	81,000	34.5206	0.00003506
*c*_8_	5	76,000	30.9263	0.00002792
*c*_9_	21	100,000	31.3032	0.0000283
*c*_10_	18	88,000	34.3504	0.00002926

First, based on the above-known information before integration, the values of ${\hat {\rm A}_{\rm s}}$ and ${\hat {\rm B}_{\rm s}}{\rm (}\infty {\rm )}$ are calculated, then the cumulative fault functions of CB-GGOM-E and CB-GGOM-S can be obtained directly. At any time t, the value of ${\hat {\rm B}_{\rm s}}{\rm (t)}$ can also be calculated, and the corresponding value of ${\hat {\rm m}_{\rm s}}{\rm (t)}$ is the prediction of the CB-GGOM for the cumulative number of system faults when the integration test time reaches *t*. As a demonstration, we present the values of ${\hat {\rm B}_{\rm s}}{\rm (t)}$ and ${\hat {\rm m}_{\rm s}}{\rm (t)}$ when *t* is taken as 1 × 10^4^, 1 × 10^5^, and 1 × 10^6^ respectively, as Table 3 shows.

**Table 3:** Parameters and functions of proposed models.

	${\hat A_s}$	${\hat B_s}(t)$	${\hat m_s}(t)$
CB-GGOM-E	34.5985	${\hat B_s}(0)= 2{ .4261} \times { 1}{{0}^{{ - 6}}}$	${\rm 34}{\rm .5985}(1 - {e^{ - {\rm 2}{\rm .4261} \times {\rm 1}{{\rm 0}^{{\rm - 6}}}t}})$
CB-GGOM-S	34.5985	${\hat B_s}(\infty ) =2{\rm .3998} \times {\rm 1}{{\rm 0}^{{ - 7}}}$	${\rm 34}{\rm .5985}(1 - {e^{ - {\rm 2}{\rm .3998} \times {\rm 1}{{\rm 0}^{{\rm - 7}}}t}})$
		${\hat B_s}(10,\!000) =2{\rm .3964} \times {\rm 1}{{\rm 0}^{{\rm - 6}}}$	0.8192
CB-GGOM	34.5985	${\hat B_s}(100,\!000)= 2{\rm .154} \times {\rm 1}{{\rm 0}^{{\rm - 6}}}$	6.7045
		${\hat B_s}(1,\!000,\!000)=9{\rm .285} \times {\rm 1}{{\rm 0}^{{\rm - 7}}}$	20.9271

**Figure 3:**
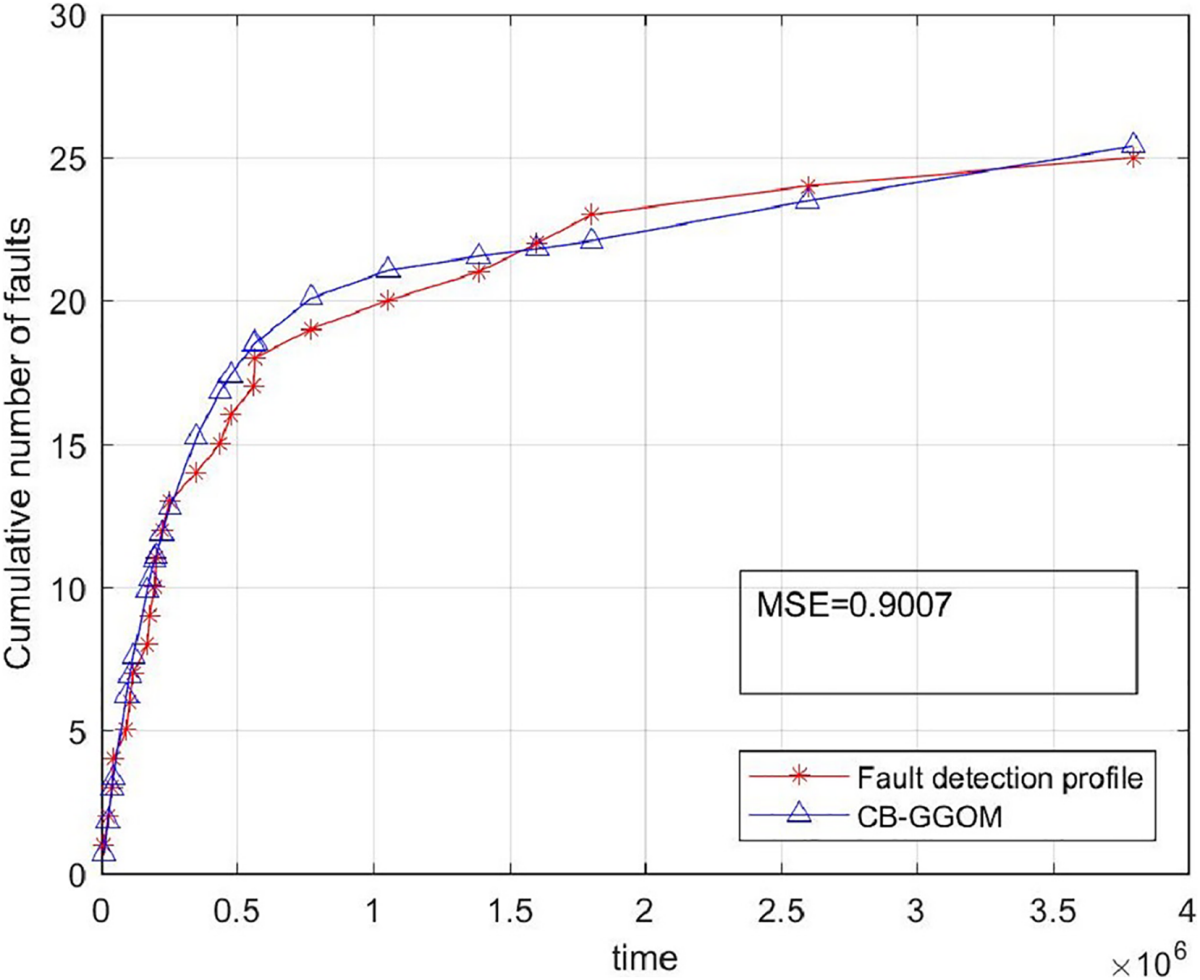
System fault growth trend predicted by CB-GGOM.

**Figure 4:**
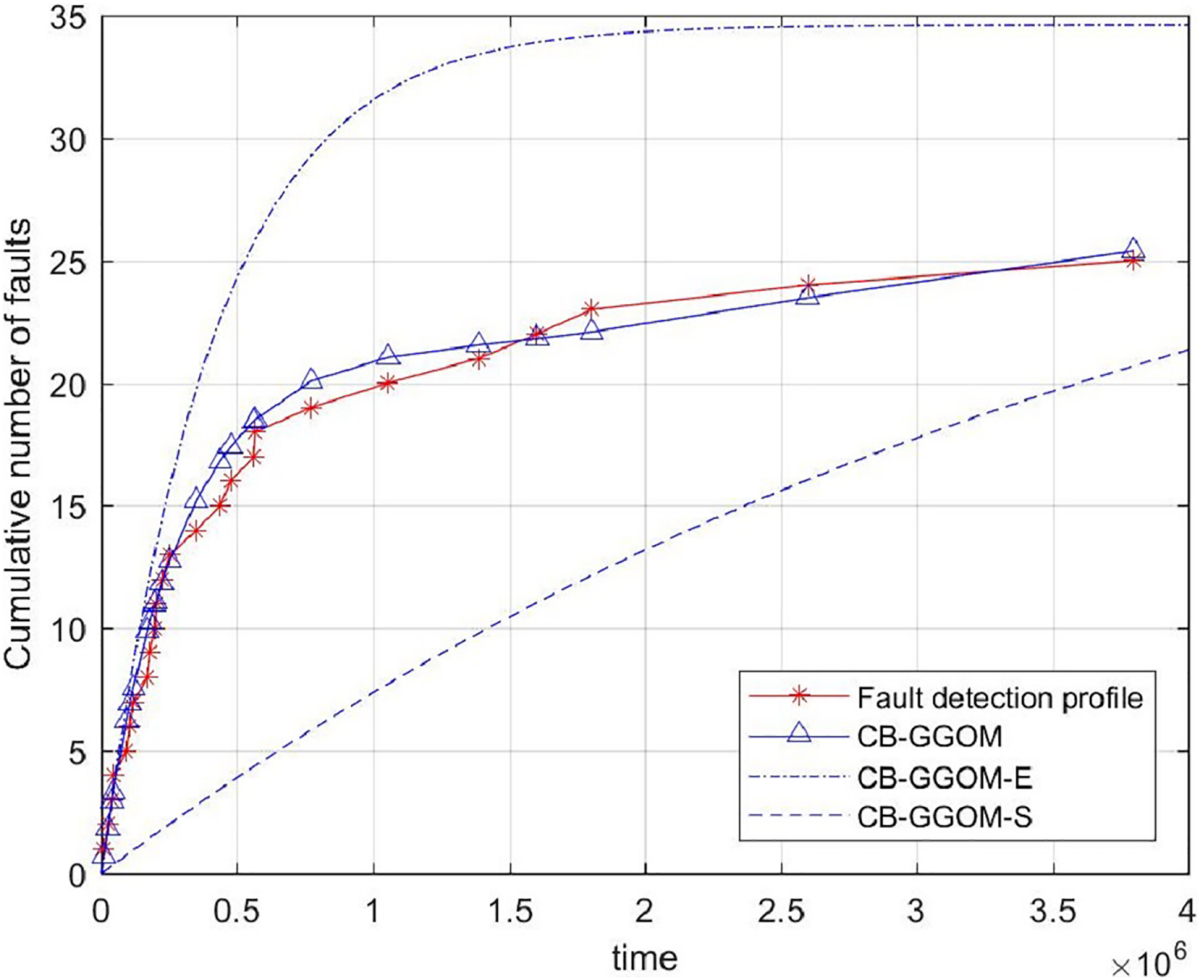
System fault growth trends predicted by CB-GGOM-E and CB-GGOM-S.

Next, we verified the prediction effects of the proposed models through simulation. We set the discrete time step of the simulation as one unit of time and the total simulation time as 4 × 10^6^ time units, then simulated the fault detection process of the system. By repeating the simulation 30 times and eliminating random errors, we obtained 25 groups of simulated system fault data (*t*_*i*_,*y*_*i*_), where *t*_*i*_ represented the simulated time of the *i*^*th*^ failure of the software system, and *y*_*i*_ represented the cumulative number of system faults at *t*_*i*_, which is a positive integer. Then, we calculated the values of ${\hat {\rm m}_{\rm s}}{\rm (}{{\rm t}_{\rm 1}}{\rm )},{\hat {\rm m}_{\rm s}}{\rm (}{{\rm t}_{\rm 2}}{\rm ), \ldots },{\hat {\rm m}_{\rm s}}{\rm (}{{\rm t}_{{\rm 25}}}{\rm )}$ of CB-GGOM at the simulated system fault time series, plotted its scatter diagram and compared it with the simulated system fault detection profile. The mean square error (MSE)

(20) $$MSE = \displaystyle{{\sum\limits_{i = 1}^n {{{[{y_i} - \hat m({t_i})]}^2}} } \over n}$$

was adopted to measure the prediction error of CB-GGOM, as Fig. 3 shows. Smaller MSE values result in better prediction effects. The images of CB-GGOM-E and CB-GGOM-S were also drawn, and are shown in Fig. 4.

Figure 3 shows that at each simulated system fault time the predicted number of cumulative faults using the CB-GGOM is relatively close to the simulation number, also the MSE is only 0.9007; according to Fig. 4, CB-GGOM-E has a good prediction performance for the growth trend of system faults in the early stage of integration testing. However, as the testing process proceeded, the growth rate gradually became faster than the simulation profile. In contrast, CB-GGOM-S generally captures the trend when the system enters a steady state. Therefore, the simulation experiments confirm the satisfactory prediction ability of CB-GGOM for the software system fault trend throughout the integration test stage. The two approximate models also have good prediction performances in the test stage they are aimed at.

**Figure 5:**
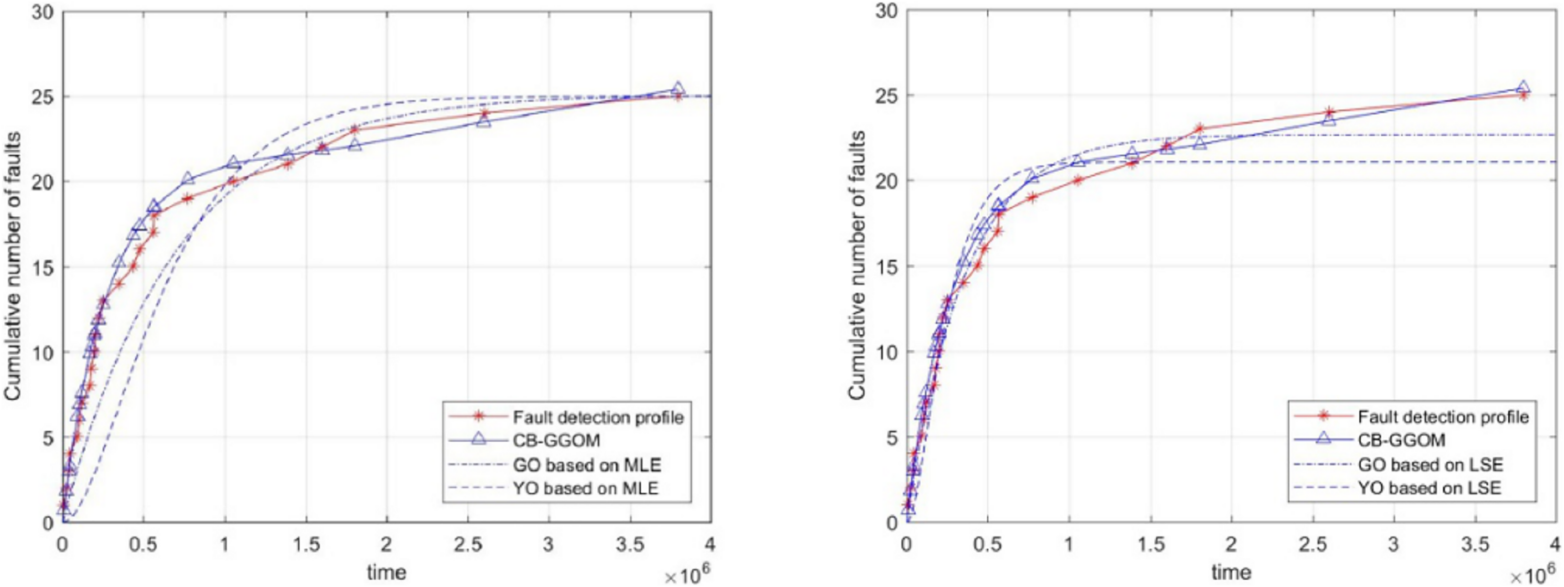
Images of the G-O model and the Y-O model.

Finally, to illustrate the significance of the CB-GGOM, we used traditional SRGMs to fit the simulated system fault data. The selected SRGMs were the G-O model and the Yamada & Ohba model (Y-O model) (Yamada, Ohba & Osaki, 1983), which is also known as the S-shaped SRGM due to the growth shape of the function curve. The parameters of the G-O model and the Y-O model were estimated respectively by two methods: maximum likelihood estimation (MLE) and least squares estimation (LSE). Their images are shown in Fig. 5. Also, the MSE was used to measure the fitting errors of the G-O model and the Y-O model, as shown in Table 4.

**Table 4:** Fitting tests of the G-O model and the Y-O model.

	Parameters estimated by MLE		MSE	Parameters estimated by LSE		MSE
	${\hat A_s}$	${\hat B_s}$		${\hat A_s}$	${\hat B_s}$	
G-O model	25.1095	2.4231 × 10^−6^	8.6131	22.6672	2.8311 × 10^−6^	0.9928
Y-O model	25.0045	2.9297 × 10^−6^	25.4932	21.0571	7.8072 × 10^−6^	3.765

In Fig. 5, the simulated growth curve of software system faults is closer to a negative exponential shape than an S shape. Also, the fitting test shows that whether MLE or LSE was adopted to estimate parameters, the error of the corresponding G-O model is smaller than that of the Y-O model. Therefore, the simulation experiments confirm the rationality of constructing the function *m*_*s*_(*t*) of the CB-GGOM in the form of the G-O model. Next, since the fitting error of the G-O model based on LSE was only 0.9928, we compared its parameter values (*i.e*., 22.6672 and 2.8311 × 10^−6^) with those of the CB-GGOM. We found that the G-O model’s parameter values had little deviation from those of CB-GGOM. However, the parameters of the G-O model were estimated based on 25 pieces of simulated system fault data, which means that the G-O model can only be used in the late stages of the integration test with 4 × 10^6^ time units. This duration is almost ten times the length of the component unit test (as per our previous statement, this is because the fault data of the software system is a small sample). In comparison, the parameters of the CB-GGOM are calculated without any integration test data so that the CB-GGOM can predict the growth trend of system faults in the design and integration stage. Therefore, the simulation experiment verifies the accuracy of CB-GGOM, and demonstrates its value in the early evaluation of software system reliability.

### Instance analysis

**Figure 6:**
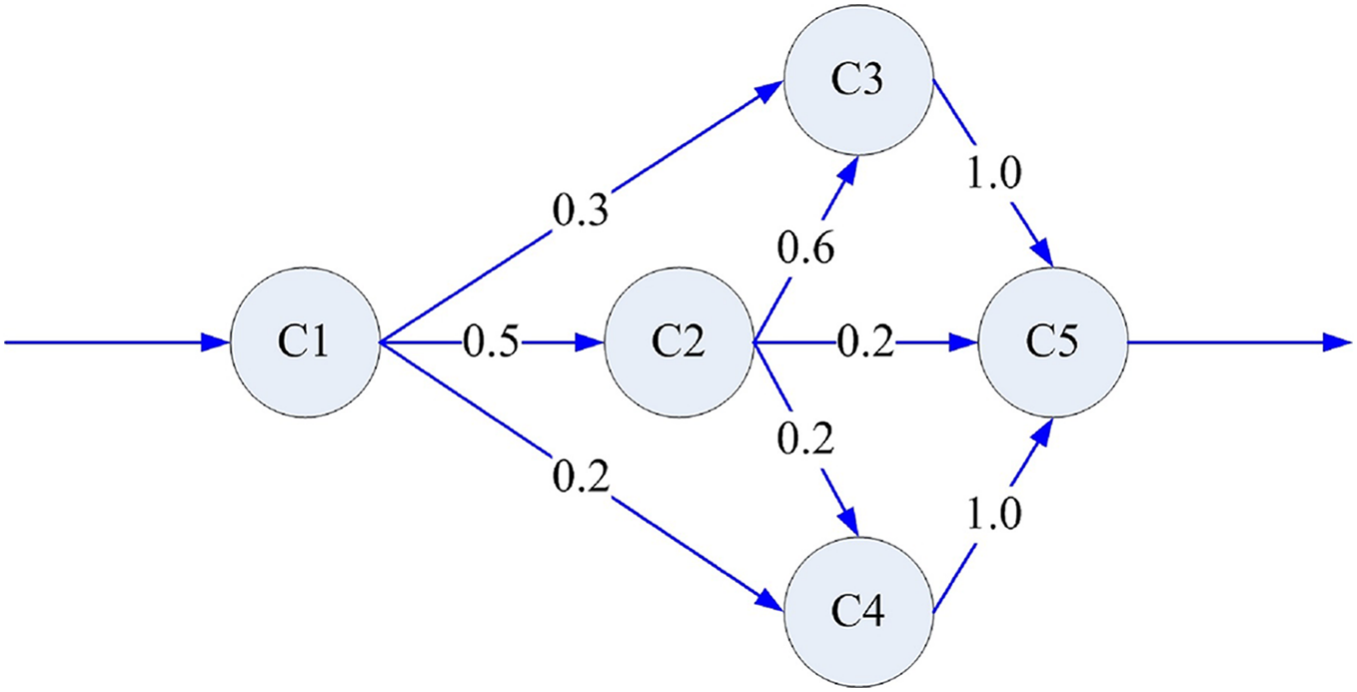
Architecture of the subsystem.

We further examined the performance of the proposed models using a subsystem of a management information system for a patent affairs process as an example (Chunyan et al., 2009). This subsystem has five components, as shown in Fig. 6. Besides, Table 5 shows the unit testing data and reliability characteristics of each component before integration. Table 6 shows the system fault times recorded during the integration testing.

**Table 5:** Unit testing data and component reliability characteristics.

Component	Average task time in one execution *τi* (s)	Last failure time of unit testing *T*_i_ (s)	Parameter estimate of the G-O model	
			*a*	*b*
*c*_1_	4.068	66,765	21.3032	2.7918 × 10^−5^
*c*_2_	65.294	82,502	24.3504	3.5056 × 10^−5^
*c*_3_	40.157	78,621	20.0043	2.9263 × 10^−5^
*c*_4_	32.652	69,075	17.8741	2.6458 × 10^−5^
*c*_5_	9.467	73,819	20.9263	1.709 × 10^−5^

**Table 6:** Integration testing data.

Cumulative number of system faults	Failure time (s)
1	1,143
2	9,286
3	26,467
4	44,319
5	52,150
6	70,359
7	89,246

By applying the data in Table 5 and Eqs. (2) and (3), we first calculated the proportion vector of the remaining faults in every component at time 0:

(21) $${\rm \vec \omega (0)\; = (0}{\rm .2137,0}{\rm .0874,0}{\rm .1297,0}{\rm .1859,0}{\rm .3834)}$$

Besides, we determined the proportion vector for the time spent on each component:

(22) $${\rm \vec \pi \; = (0}{\rm .0508,0}{\rm .4077,0}{\rm .3009,0}{\rm .1223,0}{\rm .1182)}$$

Following this, we calculated that the values of *b*_1_^*IT*^(0), *b*_2_^*IT*^(0), …, *b*_5_^*IT*^(0) are 0.303 × 10^−6^, 1.249 × 10^−6^, 1.142 × 10^−6^, 0.602 × 10^−6^, and 0.775 × 10^−6^ based on Eq. (12). Then we obtained the values of ${\hat {\rm A}_{\rm s}}$, ${\hat {\rm B}_{\rm s}}{\rm (0)}$, ${\hat {\rm B}_{\rm s}}{\rm (}\infty {\rm )}$, and ${\rm \; }{\hat {\rm B}_{\rm s}}{\rm (t)}$ in the system fault time series. For the convenience of subsequent comparisons, the parameters of the CB-NHPP model were also calculated, and are listed in Table 7.

**Table 7:** Parameters comparison.

	CB-GGOM	CB-NHPP model
${\hat A_s}$	15.4586	15.4586
	${\hat B_s}(0) = 4{\rm .0697} \times {\rm 1}{{\rm 0}^{{\rm - 6}}}$	
	${\hat B_s}({\rm 1,\!143}) = 4.0523 \times {10^{ - 6}}$	
	${\hat B_s}({\rm 9,\!286}) = 3.9321 \times {10^{ - 6}}$	
	${\hat B_s}({\rm 26,\!467}) = 3.6991 \times {10^{ - 6}}$	
${\hat B_s}(t)$	${\hat B_s}({\rm 44,\!319}) = 3.485 \times {10^{ - 6}}$	2.9774 × 10^−5^
	${\hat B_s}({\rm 52,\!150}) = 3.3994 \times {10^{ - 6}}$	
	${\hat B_s}({\rm 70,\!359}) = {\rm 3}.2184 \times {10^{ - 6}}$	
	${\hat B_s}({\rm 89,\!246}) = {\rm 3}.0549 \times {10^{ - 6}}$	
	${\hat B_s}(\infty ) = 1.4184 \times {10^{ - 6}}$	

**Figure 7:**
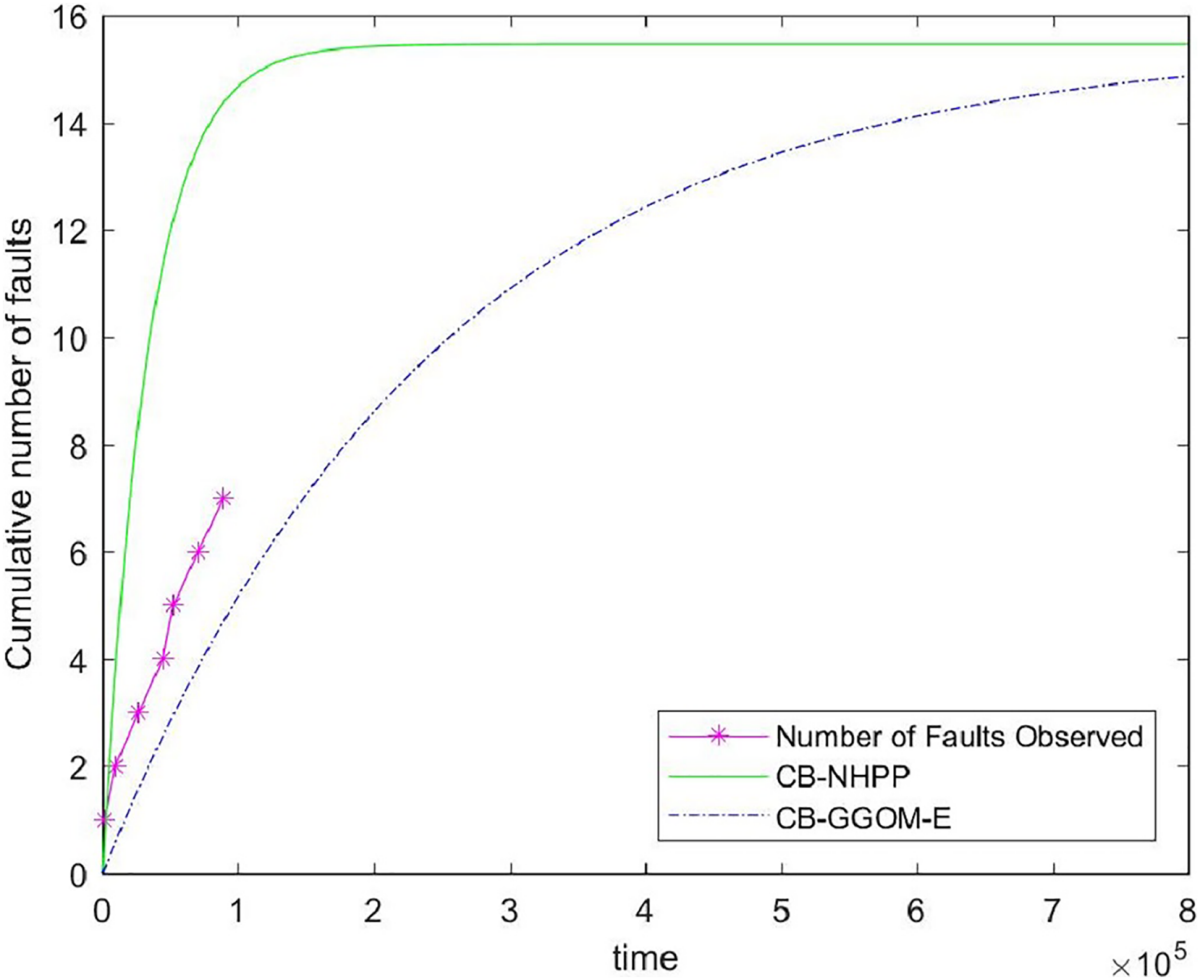
Comparison of the cumulative fault functions.

Next, we used the data in Table 6 to verify the effectiveness of the proposed models. In reality, the time spent on software testing is usually relatively short. Thus, it is advisable to choose CB-GGOM-E as the verified object. The error between the predicted values of CB-GGOM-E and the actual number of system faults was still measured using MSE, and the CB-NHPP model was also assessed, as Fig. 7 and Table 8 show.

**Table 8:** Fitting test of reliability models.

	CB-NHPP model	CB-GGOM-E
MSE	35.6453	3.0106

It can be seen from Fig. 7 that the prediction of the CB-NHPP model is close to the first two data points regarding system faults. However, with an increase in test time, the predicted trend soon deviates from the actual fault data. In comparison, CB-GGOM-E generally tracks the overall growth trend of system fault data. Error tests also show that CB-GGOM-E has superior prediction effects.

The main reason for these differences is that the fault detection rate of component *i* in the integration test stage (*i.e*., *b*_*i*_^*IT*^(*t*)) predicted by CB-GGOM-E has one more proportional factor *ω*_*i*_(*t*) than that predicted by the CB-NHPP model. According to our explanation given in Section 4, the test described by the CB-NHPP model is the process in which *n* newly developed components are independently tested. Thus, the value of ${\rm \; }\mathop \sum \limits_{{\rm i = 1}}^{\rm n} {{\pi }_{i}}{{b}_{i}}$ predicted by CB-NHPP is equivalent to the weighted average of historical fault detection levels of *n* components. With this in mind, we note that the value of ${\rm \; }\mathop \sum \limits_{{\rm i = 1}}^{\rm n} {{\pi }_{i}}{{b}_{i}}\;$ is indeed of the same order of magnitude as *b*_1_, *b*_2_, …, *b*_*n*_. In comparison, the values of ${\hat {\rm B}_{\rm s}}{\rm (t)}$ predicted by CB-GGOM are one order of magnitude lower than *b*_1_, *b*_2_, …, *b*_*n*_. It is widely known that the time spent on integration testing is actually much slower and longer than that spent on unit testing. Thus, from the quantitative analysis alone, the system fault detection level predicted by the CB-GGOM is closer to the objective reality.

Next, we analyzed the rationality of CB-GGOM theoretically. The CB-NHPP model always predicts the fault detection rate of the *i*^*th*^ component in the integration test with a constant *b*_*i*_, no matter how long the integration test lasts. Thus, if we assume that the integration test has entered a stable state, according to the prediction of the CB-NHPP model, the tester willdetect faults from a component that has no faults with the possibility *b*_*i*_. Obviously, this situation is contrary to objective fact. Therefore, the system fault detection level predicted by CB-NHPP should be faster and faster than the actual system fault detection level as the test proceeds, resulting in increasingly premature predictions for the cumulative number of system faults. In comparison, CB-GGOM uses *ω*_*i*_(*t*)*b*_*i*_ to predict the fault detection rate of component *i* in the integration test stage. Under the constant correction of *ω*_*i*_(*t*), the probability of a component without faults being detected tends to zero. Therefore, CB-GGOM can reasonably predict the growth process of system faults in the middle and late stages of the test. The findings presented in Fig. 7 and Table 8 confirm our interpretations and judgment.

**Figure 8:**
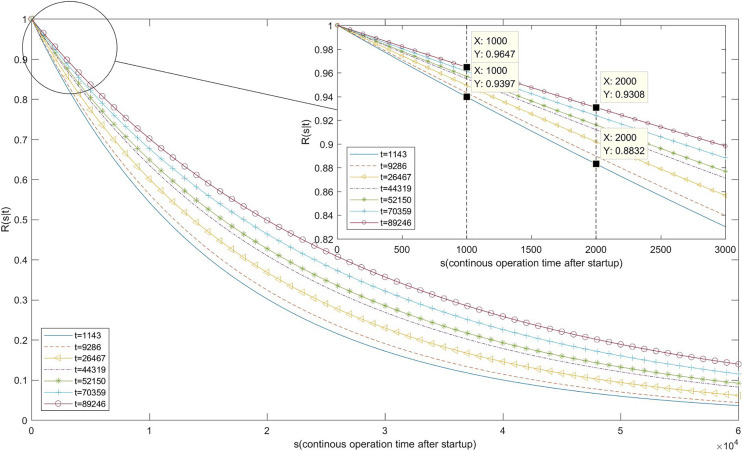
Image of reliability function.

Finally, we calculated *R*(*s*|*t*) using Eq. (18). The calculation results and image are shown in Table 9 and Fig. 8.

**Table 9:** The reliability of the software system.

*s* (second)	1,000	2,000
*R* (*s*|*t*)		
*t* (second)		
1,143	0.9397	0.8832
9,286	0.9432	0.8898
26,467	0.9496	0.9018
44,319	0.955	0.9121
52,150	0.957	0.916
70,359	0.9612	0.924
89,246	0.9647	0.9308

As Table 9 and Fig. 8 indicates, when the system fails for the first time after 1,143 s and the fault is repaired immediately, the probability of continuous operation for 1,000 s after system restart is 0.9397, and the probability of continuous operation for 2,000 s is 0.8832. As the remaining faults are removed one after the other, the reliability of the system continues to grow. When the seventh system failure occurs at 89,246 s, the probability of continuous operation for 1,000 s after fault removal and system restart increases to 0.9647, while the probability of continuous operation for 2,000 s grows to 0.9308.

## Discussion

The two main innovations of this article are the system parameter estimation models and the software system reliability growth models that incorporate these system parameters.

First, the proposed system parameter estimation models can be calculated according to the known information in the integration phase. Their main function in this article is to serve as the input of the software system reliability growth models. Due to the incorporation of system parameters, the proposed growth models can evaluate and predict the reliability growth trends of the software system during the integration testing process without integration test data. Therefore, the reliability growth models are useful for software system developers to grasp the maturities of the current design scheme and test scheme, thereby assessing software development costs and planning the project schedule.

Another advantage of the proposed system parameter estimation models is that they are expressed by the sum of the product of the weight factors. To focus on the topic of software system reliability evaluation, we only discuss the interpretability of these factors of the proposed models and do not dig into their other values in this article. Actually, accurate identification of the weakest components of the software system in the design phase is also a beneficial work, because this identification can helps the software system developers to formulate targeted prevention strategies and eliminate potential hazards in the design stage and guarantee the quality of the software system economically and efficiently (Li et al., 2017; Kaur, Singh & Singh, 2015). However, even if a component is fully tested before delivery, it may also gain more testing time resources due to higher call frequency and longer operation time during the integration test process. As a result, internal residual faults are exposed first, thus becoming the weakest component of the software system. The system parameter estimation models proposed in this article include weight factors that reveal the above objective laws and also quantify the impact of each factor. In our opinion, they could provide a reference for inference of weakest components and optimization of software system testing strategies.

We can take the instance in Part B of Section 5 as a concise demonstration. First, by applying ${\rm \vec \omega }$(0) in Eq. (21), we determine that the proportion of the residual faults in each component at the initial time is c5 > c1 > c4 > c3 > c2 in descending order, and the proportion of c5 is four times that of c2. Besides, using ${\vec \pi }$ in Eq. (22) reveals that the proportion of the average tested time of each component in the integration test is c2 > c3 > c4 > c5 > c1 in descending order, and c2 is 8 times larger than c1. Moreover, using *b*_*i*_^*IT*^(0), we can predict that the probability of faults detected in the component at the beginning of the integration test is c2 > c3 > c5 > c4 > c1 in descending order, and the probabilities of c2 and c3 are almost four times that of c1. Since the overall probability of faults being detected in the software system at time *t* is dependent on the probability of faults being detected in each component, the component with the highest *b*_*i*_^*IT*^(0) value is the one with the highest probability of faults being detected first at the initial stage of the integration test. From this, we know that c2 and c3 are the two most fully tested components before delivery, but they may be the two components that are most likely to fail first in the early phase of the integration test due to the longer test time allocated in integration testing. Therefore, c2 and c3 should be the focus of software system developers. Based on this inference we can provide further suggestions for the optimization of software system testing strategies. We know that within the specified time interval, smaller fault detection probability reflects a more reliable software system. For the *B*_*s*_(*t*) proposed in this article, the value of *b*_*i*_ is determined by the unit testing environment before component delivery, and the value of *π*_*i*_ is determined by the user’s operating habits and the functional attributes of the components, that is, these two factors are not easy to be modified by the software system developers. Therefore, an effective method is to strengthen testing for weakest component first and then implement integration testing according to the original scheme. This testing strategy actually reduces the value of *B*_*s*_(0) by only reducing the value of *ω*_*i*_(0) of the weakest component. Theoretically, it can maximize the level of software system reliability without modifying the structural design and the original integration test scheme, so it is an operable and low-cost testing method.

## Future work

In this article, we propose a reliability growth model named CB-GGOM, as well as two approximate models, CB-GGOM-E and CB-GGOM-S, for a software system whose component reliability follows the G-O model. The G-O model assumes that once a fault is detected, it immediately causes software failure. Thus, the models proposed in this article also regard that once a fault in a component is detected, the component and the entire software system both immediately fail. This assumption is rigorous and does not correspond exactly with reality, so the evaluation results of the proposed models are conservative. Fortunately, some SRGMs of single software that consider failure delay and degraded operation have been studied, and many of them have been enhanced according to the G-O model. Therefore, we can improve the CB-GGOM based on these research results, to obtain evaluation results that are closer to reality.

To facilitate the establishment of the mathematical model, we propose some assumptions in the third section. The analyses and citations in this section are to illustrate that the proposed assumptions are derived from the engineering approximation, so the deviation between the assumption and the actual situation is not too large to be accepted. Besides, there are some situations that are not covered by the proposed models, such as the constraints of the test cost, the introduction of new faults during the debugging process, and the adjustment of test strategies caused by the participation of multiple debugging personnel. The solutions of these situations usually involve other theories, relevant references and integration test data, so they need to be explored in the subsequent research, and reasonable engineering approximation is still an effective means for subsequent model construction.

To verify the effectiveness of the proposed models, we use mean square error to measure the deviation between the cumulative fault function and the test data in Section 5. Since the cumulative fault function is also an increasing function, it is beneficial to know its supersaturation to the horizontal asymptote in the Hausdorff metric. This metric allows researchers to choose an appropriate model for approximating specific data from different branches of scientific knowledge (Pavlov et al., 2018; Kyurkchiev & Markov, 2015). However, it is not easy to obtain the Hausdorff metric of the cumulative fault function proposed in this article, because the mathematical expression of the proposed function is composed of a sum function and an exponential function, which is relatively complex. Nevertheless, the cumulative fault function based on NHPP theory is still a mean function, and several scholars have discussed the Hausdorff approximation of the mean function by performing polynomial variable transfer (Neha, Aggarwal & Jaiswal, 2022; Kyurkchiev, Iliev & Rahnev, 2020), which facilitates our in-depth discussion. Due to length limitations, we will continue this discussion in future papers to enhance the applicability of our proposed models.

In Section 6, we provide a tentative discussion and a brief demonstration of how the proposed system parameter estimation models could be applied to infer the weakest components of the software system and optimize testing strategies. Certainly, the validity and rationality of these inferences and suggestions should be further verified in diverse situations. Assorted simulation techniques should also be adopted to substantiate the value and contribution of the proposed models. We think that this application of system parameter estimation models should be explored as a special topic in the future.

## Supplemental Information

10.7717/peerj-cs.1247/supp-1 Supplemental Information 1 Code.

## Additional Information and Declarations

### Data Availability

The following information was supplied regarding data availability: The code is available in the Supplemental File.
